# Genome-Wide Microsatellites in *Acanthopagrus latus*: Development, Distribution, Characterization, and Polymorphism

**DOI:** 10.3390/ani14243709

**Published:** 2024-12-23

**Authors:** Chao Peng, Congqiang Luo, Guangqing Xiang, Jiezhen Huang, Liye Shao, Haihong Huang, Sigang Fan

**Affiliations:** 1 Changde Key Innovation Team for Wetland Biology and Environmental Ecology, Hunan Provincial Key Laboratory for Molecular Immunity Technology of Aquatic Animal Diseases, College of Life and Environmental Science, Hunan University of Arts and Science, Changde 415000, China; pengchao@huas.edu.cn (C.P.); 15602258587@163.com (C.L.); shaoly99@163.com (L.S.); shinkanh@nwsuaf.edu.cn (H.H.); 2 Longshan Animal Husbandry and Fisheries Affairs Center, Xiangxi 416800, China; lsxscz6198@163.com (G.X.); blackball@yeah.net (J.H.); 3 Guangdong Provincial Key Laboratory of Fishery Ecology and Environment, South China Sea Fisheries Research Institute, Chinese Academy of Fishery Sciences, Guangzhou 510300, China

**Keywords:** genome-wide, microsatellite, *Acanthopagrus latus*, GO, KEGG

## Abstract

The yellowfin seabream (*Acanthopagrus latus*) is an economically important commercial mariculture fish in China and Southeast Asia. Only a few simple sequence repeats (SSRs) of *A. latus* have been isolated and reported, which has hindered breeding progress. A total of 318,862 SSRs were isolated and characterized from the *A. latus* genome in this study. All SSRs were 9,069,670 bp in length, accounting for 1.32% of the genome. The density and frequency of SSRs were 468.40 loci/Mb and 13,323.19 bp/Mb, respectively. The major SSRs were dinucleotides (accounting for 76.92%), followed by trinucleotides (15.75%). The most abundant SSR motif was (AC)n (168,390, accounting for 53%), with the highest frequency (245.78 loci/Mb) and density (7304.18 bp/Mb). Most SSRs were located in non-coding regions, such as intergenic regions (34.54%) and introns (56.91%). SSR-containing exons were distributed into 51 gene ontology (GO) terms and significantly enriched in immunity- and growth-related pathways. A total of 217,791 SSR markers were successfully designed. Nine SSR markers were amplified in 29 *A. latus* individuals, and eight of them possess high polymorphism. The cross-species transferability of 33 out of the 37 tested loci were successfully amplified in *Acanthopagrus schlegelii*. These results lay the foundation for the molecular marker-assisted breeding and genetic information assessment of *A. latus*.

## Simple Summary

The yellowfin seabream *Acanthopagrus latus* is an economically important marine fish in China and Southeast Asia. Currently, only a few simple sequence repeats (SSRs) have been isolated from the *A. latus* genome. In this study, genome-wide SSRs were screened and characterized in *A. latus* for the first time. The total length, density, frequency, and distribution of SSRs were analyzed. SSR-containing exons were enriched in gene ontology (GO) and KEGG. Some polymorphismic SSR markers were obtained. These results provided enough data for breeding programs and genetic evaluations of *A. latus*.

## 1. Introduction

The yellowfin seabream *Acanthopagrus latus* (Houttuyn, 1782), belonging to the Sparidae family, is a reef-dwelling species that is widely distributed in the warm coastal waters of the Indo-West Pacific, such as the coasts of Japan, Korea, China, and Vietnam [1,2]. *A. latus* is an economically important mariculture fish species. The production of seabream was 147 thousand tons in China in 2023 [3]. *A. latus* is always cultured in pond and net cages in South China and Southeast Asia [4,5]. Because of its good meat quality and delicious taste, *A. latus* is a popular seafood across the world [1].

Microsatellites, also known as simple sequence repeats (SSRs), are a group of DNA sequences consisting of tandem repeated units (2–6 bp) [6]. Due to the characteristics of co-domain inheritance, high polymorphism, abundance, and random distribution in the genome, microsatellites have proven to be a useful tool in the research of aquaculture species, including species identification, genetic structure analysis, marker-assisted selection, parentage determination, and quantitative trait loci (QTL) mapping [7,8,9,10,11,12]. For example, the parentage assignment of *Monopterus albus* was established using 16 high polymorphic SSR markers [9]. More than 1000 SSR markers were used to construct a genetic linkage map, and a quantitative trait locus (QTL) associated with flowering time was found [10]. A genetic linkage map of sweet potato was constructed using SSR markers, and seven QTLs associated with resistance to root rot were detected [11]. Sixteen SSR markers were reliable tools for identification and phylogenetic analyses across deer species [12]. Although microsatellites are located in various genome regions, the regions flanking microsatellites are conserved and could serve as templates to design locus-specific SSR markers. To date, limited SSR markers have been developed from *A. latus* [13,14]. More polymorphic SSR markers are needed to satisfy the increasing needs of molecular breeders and geneticists in achieving more precise marker-assisted selection.

Recently, with the development of next-generation sequencing technologies, numerous genomes have been sequenced and assembled in an efficient and cost-effective manner [15]. Higher numbers of SSRs were rapidly and effectively isolated and developed from genome sequences [16,17,18,19,20]. For example, Jiang et al. [16] examined millions of genomic SSRs from the draft genome of 30 marine animals and found many differences in SSR characteristics and two taxon-specific SSR types. Luo et al. [17] detected many sets of SSR markers from the *Acanthopagrus schlegelii* genome and provided a cost- and resource-efficient method of whole-genome resequencing combined with HipSTR analysis to examine the polymorphic loci. Fan et al. [18] isolated 220,709 SSRs from the genome of *Lateolabrax maculatus* and demonstrated that the total length of the SSRs were positively correlated with the genome size. The genome sequences of *A. latus* (GCA_904848185.1) at the chromosome level were reported [21], which provided enough data to screen and develop SSRs of *A. latus*.

In this study, we screened genome-wide SSRs from *A. latus* genome and characterized the number, frequency, distribution, and types of repeat motifs. Genes containing microsatellites were analyzed by gene ontology (GO) and the Kyoto Encyclopedia of Genes and Genomes (KEGG). Furthermore, more than 38 polymorphic and stable SSR markers were developed. This study provides novel insights into SSRs in *A. latus* genome, which may be useful for breeding projects of *A. latus* in the future.

## 2. Materials and Methods

### 2.1. Sample Collection and DNA Extraction

Twenty-nine wild *A. latus* individuals were caught in Hailing Bay, Yangjiang (Guangdong Province, China), in April 2024. Each muscle was cut and stored in liquid nitrogen until use. High-quality genomic DNA was extracted from the muscle tissue using the EasyPure^®^ Marine Animal Genomic DNA Kit (TransGen Biotech, Beijing, China), following the manufacturer’s protocol. The DNA integrity and concentration were examined with agarose gel (1.2%) electrophoresis and NanoDrop 2000 (Thermo Fisher Scientific, Waltham, MA, USA), respectively.

### 2.2. Genome-Wide Microsatellites Mining

The *A. latus* genome (GCA_904848185.1) reported by Lu et al. [21] was downloaded from NCBI (http://www.ncbi.nlm.nih.gov/ (accessed on 9 August 2024)). SSR loci were scanned from the genome of *A. latus* by the MISA program (https://webblast.ipk-gatersleben.de/misa/ (accessed on 9 August 2024)) [22]. The parameters were listed as follows: for dinucleotide repeats motifs, repeat times ≥ 6; for tri-, quad-, penta- and hexa-nucleotide repeat motifs, repeat times ≥ 5; for compound microsatellites, the interval between two repeats motifs < 100 nt.

When checking different SSR motifs, repeats with unit patterns that were circular permutations and reverse complements were considered as one type for statistical analysis. For example, the numbers of the motif ACT, CTA, and TAC microsatellites were added to the ACT microsatellites, and ACT also denotes TGA, GAT, and ATG on the complementary strand. The shortest basic sequence was treated as the motif of the SSR with any number of repeats of the basic sequence. For example, the motif (ATAT)_8_ is (AT)_16_, and thus the (TCTC)_10_ was treated as (TC)_20_.

The distribution of SSRs in exonic, intronic, and intergenic regions were identified using Perl scripts based on the genome annotation files of GCA_904848185.1 [21].

### 2.3. Characteristics of SSRs in the A. latus Genome and Chromosome

The abundance, frequency, and density of SSRs in each chromosome and whole genome were calculated and analyzed by Excel 2019 (Microsoft, Redmond, Washington, DC, USA).

The length of each SSR = the length of SSR repeat × repeats number. For example, the length of SSR (AC)_12_ = 2 × 12 = 24 bp. The sum of each SSR length was the total length of all SSRs.

The frequency of SSRs = the total length of chromosome or genome (Mb)/the number of SSRs in the chromosome or genome.

The density of SSRs = the total length of the chromosome or genome (Mb)/the length of SSRs in the chromosome or genome.

The relationship between genome/chromosome size and SSR length/abundance/density was tested using the Pearson test in SPSS 19.0 (IBM, Armonk, New York, NY, USA).

### 2.4. GO and KEGG Enrichment

All of the exon region sequences contained microsatellites were analyzed by Blast2GO and WEGO for GO enrichment [23,24]. Pathway assignments were mapped based on the KEGG database (http://www.genome.ad.jp/kegg/ (accessed on 9 August 2024)).

### 2.5. Primer Designing and Validation

SSR primers were designed from the microsatellite flanking sequences using the Primer3 program (http://primer3.org/ (accessed on 9 August 2024)). The parameters were listed as follows: primer length 18–30 bp, melting temperature (Tm) 50 °C–60 °C, GC content 40–60%, PCR product size 100–300 bp. Forty-seven pairs of SSR primers were chosen randomly and synthesized by Sangon Biotech (Shanghai, China) (Table S1). The primers were examined firstly with polymerase chain reaction (PCR) in two *A. latus* individuals. Each 20 µL reaction contained 50 ng of DNA, 1 unit of ExTaq polymerase (Takara, Shiga, Japan), 1 × ExTaq PCR buffer (Mg^2+^ plus), 0.2 mM dNTPs, and 0.2 mM of each primer. The PCR program was as follows: initial denaturation at 94 °C for 5 min, followed by 35 cycles of 94 °C for 30 s, 53 °C for 30 s, and 72 °C for 30 s, with a final extension at 72 °C for 5 min. PCR was carried out using a Thermal Cycler T100 (BioRad Laboratories, Hercules, CA, USA), and the PCR products were assessed using agarose gel (1%) electrophoresis. The primers which produced expected sizes were chosen for further research. Forward primers were fluorescence-labeled with 6-Carboxyfluorescein (6-FAM) at the 5′ end. After fluorescence PCR amplification as described above, the products were subjected to capillary electrophoresis (CE) using an ABI 3730XL system (Applied Biosystems, Foster City, CA, USA), and output data were analyzed by GeneMapper v6.0 (www.thermofisher.cn/order/catalog/product/4475073?SID=fr-cesoftware-1/ (accessed on 19 August 2024)).

Nine polymorphic SSR markers (Hqd1, Hqd2, Hqd3, Hqd6, Hqd8, Hqd12, Hqd14, Hqd32, and Hqd33) were chosen randomly and used to investigate the genetic diversity of 29 wild *A. latus* individuals from Yangjiang (Table S1). The number of alleles (*Na*), number of effective alleles (*Ne*), expected (*He*) and observed (*Ho*) heterozygosities, and Shannon’s Information index (*I*) were performed with Popgene 1.32 [25]. Polymorphism information content (PIC) was carried out using PowerMarker v.3.25 software [26].

### 2.6. SSR Markers Cross-Species Transferability in Acanthopagrus

The cross-species transferability of the SSR markers of *A. latus* was tested in *A. schlegelii*. The conditions for DNA extraction and PCR amplification were as described above. Thirty-seven SSRs primer pairs, which amplified successfully in *A. latus* (Table S1), were chosen. The loci with at least one band of the expected size were considered as transferable.

## 3. Results

### 3.1. Features of the SSRs in the A. latus Genome

The total length of the *A. latus* genome, consisting of 24 chromosomes, was 680.74 Mb (Table S2). We detected 318,862 SSRs in the *A. latus* genome (Table 1 and Table S2). The average distance between each SSR was 2.15 Kb. More than 92,089 compound microsatellites (accounting for 28.89%) were identified (Table S2). The total length of SSRs in the genome was 9,069,670 bp, accounting for 1.32% of the whole genome length (Table 1 and Table S2). The frequency and density of SSRs were 468.41 loci/Mb and 13,237.93 bp/Mb, respectively (Table 1 and Table S2).

**Figure 1:**
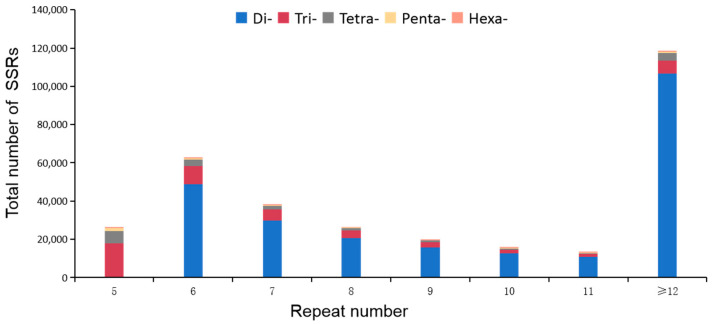
Distribution of all SSR motif repeat numbers in *A. latus* genome.

A total of 271 SSR repeat types (motifs) were found in the *A. latus* genome (Table S3). Of these SSRs, there were 4, 10, 32, 94, and 131 types of dinucleotide, trinucleotide, tetranucleotide, pentanucleotide, and hexanucleotide motifs, respectively (Table S3). The distribution of SSRs in relation to the number of repeat units is shown in Figure 1. The repeat number of all of these motifs ranged from 5 to 319, mainly between 5 and 12 (>10,000) (Figure 1).

Dinucleotide repeats (245,271, 76.92%) were the most abundant, followed by trinucleotide (50,220, 15.75%), tetranucleotide (18,107, 5.68%), pentanucleotide (4108, 1.9%), and hexanucleotide (1156, 0.36%) SSRs (Table 1). The highest frequency repeat was dinucleotide (357.99 loci/Mb), followed by trinucleotide (73.30 loci/Mb) and tetranucleotide (26.43 loci/Mb) (Table 1).

**Figure 2:**
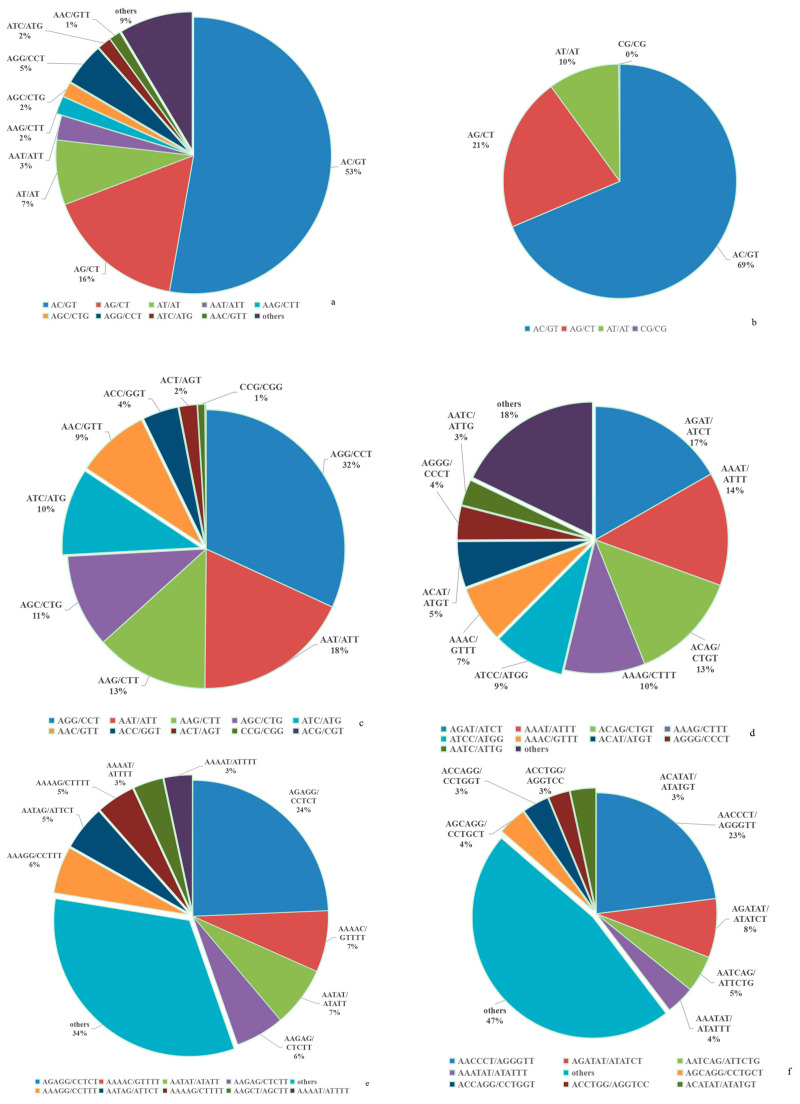
Distribution of SSR repeats in *A. latus* genome. (**a**) All SSR repeats in genome. (**b**) Dinucleotide repeat. (**c**) Trinucleotide repeat. (**d**) Tetranucleotide repeat. (**e**) Pentanucleotide repeat. (**f**) Hexanucleotide repeat.

The 10 most abundant SSR repeat motifs are listed in Table 2 and Figure 2a. They contained three dinucleotide types, six trinucleotide types, and one tetranucleotide type. The most abundant SSR type was the AC repeat (168,390, accounting for 53%), which had the highest frequency (245.78 loci/Mb) and density (7304.18 bp/Mb). The second and third most abundant SSR repeats were AG (52,305, 76.34 loci/Mb) and AT (24,152, 35.25 loci/Mb), respectively. Only 424 CG repeats were detected, which was the least abundant dinucleotide (Figure 2b). AGG had the highest frequency (23.34 loci/Mb) and density (505.54 bp/Mb) in trinucleotide repeats, followed by AAT (13.43 loci/Mb, 426.77 bp/Mb), AAG (9.65 loci/Mb, 245.21 bp/Mb), and AGC (7.95 loci/Mb, 162.52 bp/Mb). The proportion of the four trinucleotide repeats was 74% in the category of trinucleotide (Figure 2c). AGAT was the only type of tetranucleotide in the top-10 abundant motif categories, with a frequency of 4.43 loci/Mb (Table 2). The proportion of AGAT was 17% for tetranucleotides, followed by AAAT (14%) and ACAG (13%) (Figure 2d). With respect to pentanucleotides, the most abundant repeat type was AGAGG, which accounted for 24% (Figure 2e). AACCCT accounted for 23% of hexanucleotides (Figure 2f).

**Figure 3:**
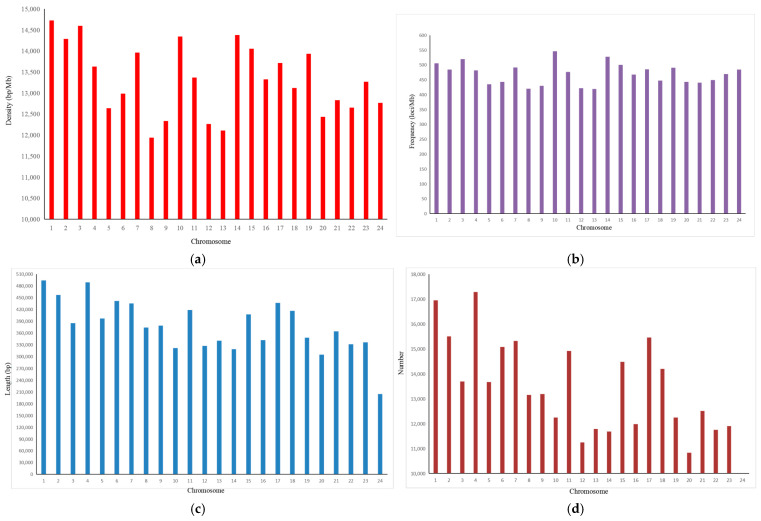
Chromosome-wide distribution of SSRs in *A. latus* genome. (**a**) Density of SSRs; (**b**) frequency of SSRs; (**c**) length of SSRs; (**d**) number of SSRs.

The characteristics and distribution of SSRs in the 24 chromosomes of *A. latus* are presented in Figure 3 and Table S2. The average SSR density of 24 chromosomes was 13,319.79 bp/Mb. The density of the SSRs was highest in chromosome 1 (14,728.51 bp/Mb), followed by chromosome 3 (14,600.15 bp/Mb) and chromosome 14 (14,380.59 bp/Mb). The lowest frequency of SSRs was 11,939.70 bp/Mb in chromosome 8 (Figure 3a; Table S2). The average frequency of SSRs was 469.89 loci/Mb (Table S2). The frequency of SSRs was highest in chromosome 10 (546.36 loci/Mb), followed by chromosome 14 (527.34 loci/Mb) and chromosome 3 (519.62 loci/Mb) (Figure 3b; Table S2). The frequency of SSRs was lowest in chromosome 13 (419.53 loci/Mb) (Figure 3b; Table S2). The maximum length of SSRs was shown on chromosome 1 (493,815 bp), followed by chromosome 4 (489,454 bp) and chromosome 2 (457,348 bp) (Figure 3c; Table S2). The minimum length of SSRs was presented on chromosome 24 (204,844 bp). The average length of SSRs in the chromosomes was 377,903 bp (Table S2). The largest number of SSRs was 17,288, presented on chromosome 4, followed by chromosome 1 (16,946) and chromosome 2 (15,498) (Figure 3d; Table S2). The minimum number of SSRs was presented on chromosome 24 (7768). The average number of SSRs in the chromosomes was 13,286 (Table S2).

**Figure 4:**
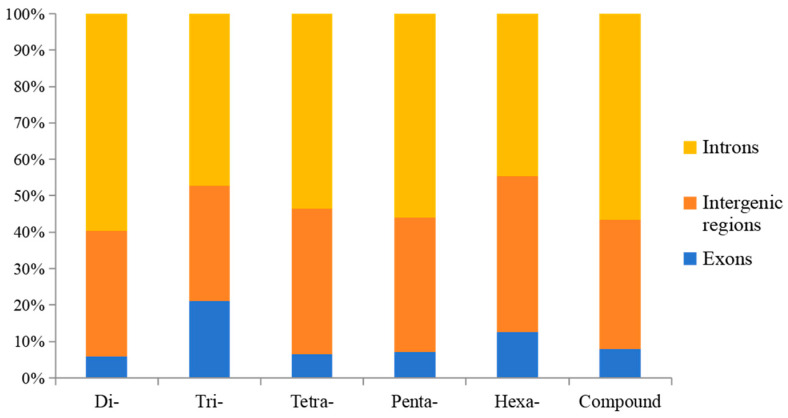
Percentage of SSR repeat numbers in different regions of *A. latus* genome.

**Figure 5:**
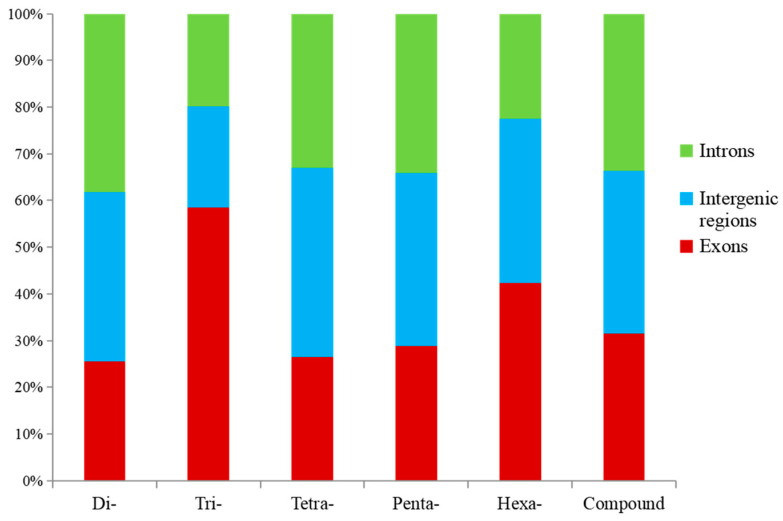
Relative proportion of SSRs in different genomic regions of *A. latus*.

More than 128,939 SSRs (accounting for 56.91%) were located in intronic regions, followed by intergenic regions (78,256, 34.54%) and exonic regions (19,378, 8.55%) (Table 3). Dinucleotide SSRs were the major type in these regions (132,339), followed by compound SSRs (47,371) and trinucleotide SSRs (31,651) (Table 3). The largest proportion of each SSR repeat was in intronic regions (44.67–59.69%), followed by the intergenic (31.68–42.68%) and exonic (5.98–21.03%) regions (Figure 4). More than 7909 dinucleotide SSRs were found in exons, followed by trinucleotide (6655) and compound (3776) SSRs (Table 3). The relative percentage of trinucleotide SSRs located in exons was highest in all SSR repeats, followed by hexanucleotide SSRs (Figure 5).

### 3.2. GO and KEGG Pathway Analyses

**Figure 6:**
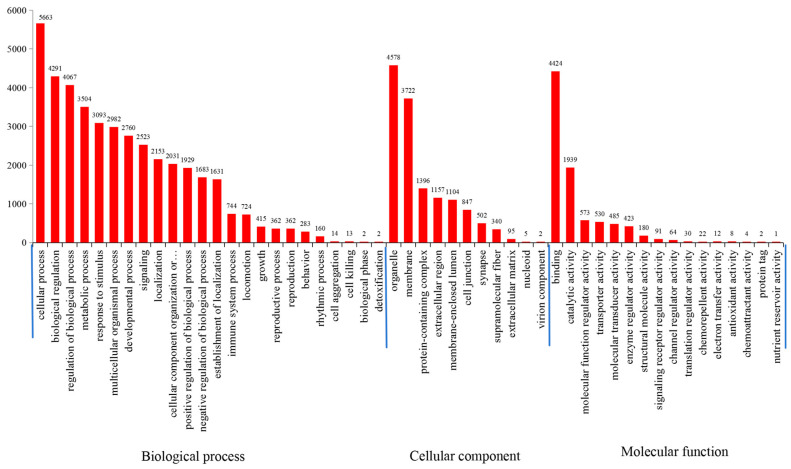
GO classifications of SSR-containing exons.

GO enrichment analyses revealed the SSR-containing exons to be distributed into 51 GO terms from three major categories: (i) biological process (6129; 32.81%), (ii) cellular component (6853; 36.68%), and (iii) molecular function (5701; 30.51%) (Figure 6). In the cellular-component category, the most abundant terms associated with genes containing SSRs were “organelle” (GO:0043226), “membrane” (GO:0016020), and “protein-containing complex” (GO:0032991) (Figure 6). “Binding” (GO:0005488) and “catalytic activity” (GO:0003824) constituted major portions under the molecular-function category (Figure 6). In the biological-processes category, most genes were enriched in “cellular process” (GO:0009987), “biological regulation” (GO:0065007), and “regulation of biological process” (GO:0050789) (Figure 6). Some expanded genes enriched in GO annotations, such as “intracellular anatomical structure” (GO:0005622), “nucleic acid binding” (GO:0003676), “zinc ion binding” (GO:0008270), and others [4], were also found (Table S4). In addition, “arrestin domain containing protein 3” (ARRDC3) and “solute carrier family 12 member” (SLC12A) were detected in GO annotations (Table S4).

**Figure 7:**
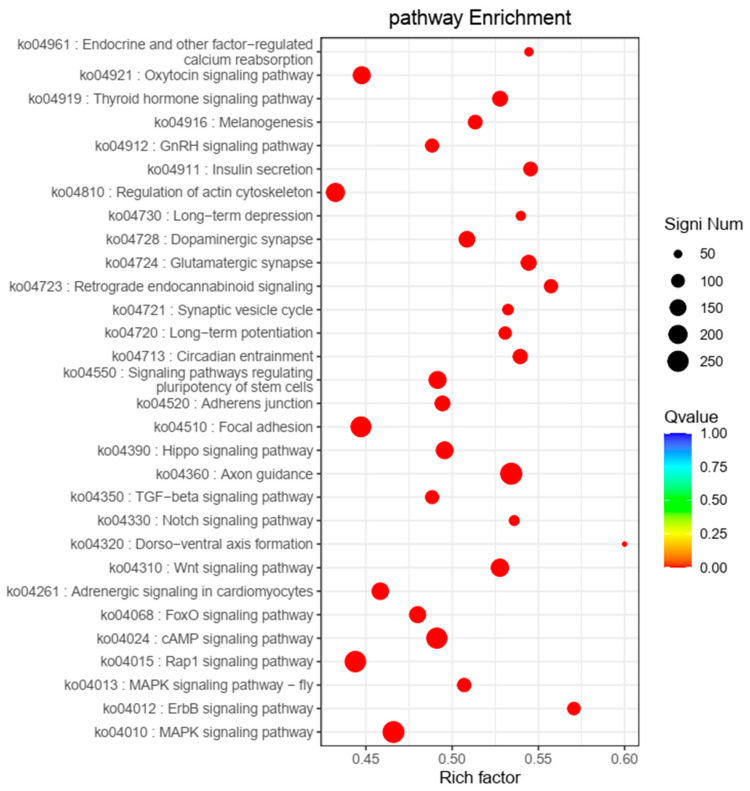
Bubble diagram of the top 30 KEGG pathways enriched by exons contained SSR.

We discovered that 4368 SSR-containing exons were assigned to 258 KEGG pathways, such as “cellular processes” (1680 SSRs; 18.19%), “environmental information processing” (2488; 26.94%), “genetic information processing” (642; 6.95%), “metabolism” (882; 9.55%), and “organismal systems” (3545; 38.38%). Several pathways associated with immunity and growth were enriched in SSR-containing exons: “Hippo signaling pathway”, “TGF-beta signaling pathway”, “insulin secretion”, “regulation of actin cytoskeleton”, “thyroid hormone signaling pathway”, and “Wnt signaling pathway” (Figure 7).

### 3.3. Development of SSR Markers

**Figure 8:**
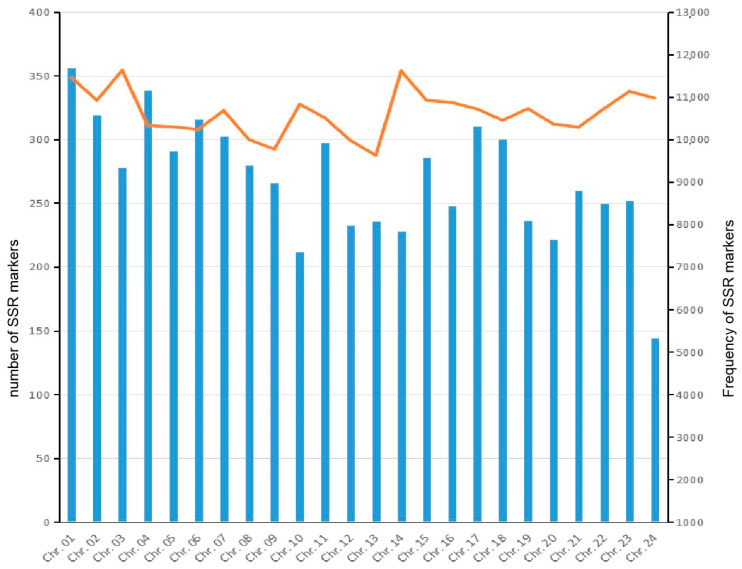
Distribution of the number (shown as columnar) and frequency (shown as broken line) of SSR markers in each chromosome of *A. latus*.

A total of 217,791 SSR markers were developed from the *A. latus* genome, with an average of 9075 in each chromosome (Table S5). The largest number of SSR markers (11,686) was found on chromosome 1, followed by chromosome 4 (11,165) and chromosome 2 (10,586) (Table S5; Figure 8). The lowest number of SSR markers (5333) was detected on chromosome 24 (Table S5; Figure 8). The frequency of SSR markers ranged from 287.40 per Mb on chromosome 13 to 354.26 per Mb on chromosome 3, with an average of 319.93 per Mb (Table S5; Figure 8). The major motif of SSR markers was dinucleotide, accounting for 57.88% to 63.36% in each chromosome, followed by trinucleotide (12.40–18.30%), tetranucleotide (3.98–6.14%), pentanucleotide (0.68–1.67%), and hexanucleotide (0.12–0.51%) (Table S5). The compound SSR markers ranged from 17.66% to 20.37% (Table S5).

### 3.4. Evaluation of the Polymorphism of SSR Markers

A total of 47 pairs of primers were synthesized to evaluate the polymorphism of SSR markers chosen randomly. Of the total primer sets tested, the PCR products from 38 pairs of primers (80.85%) that elicited a clear and reproducible band were checked by agarose-gel electrophoresis (Table S1). The remaining nine SSR primer pairs showed ambiguous and/or non-specific amplification bands.

Nine out of 37 SSR markers were characterized by capillary electrophoresis in 29 wild *A. latus* individuals (Table S1, Table 4). A total of 62 alleles were detected in nine SSR markers (Table 4). The number of *Na* ranged from three to ten (average: 6.8889), and *Ne* ranged from 5.2399 to 1.9157 (average: 3.8792). *Ho* and *He* varied from 0.1034 to 0.5172 (average: 0.2759) and 0.4864 to 0.8234 (average: 0.7308), respectively. *I* ranged from 0.8398 to 1.7962 (average: 1.5037). The PIC ranged from 0.4145 to 0.7843 (average: 0.6775). Eight SSR markers had high information (PIC > 0.5).

### 3.5. Cross-Species Development of SSR Markers in A. schlegelii

Of 37 microsatellite markers, 33 were amplified in *A. schlegelii*, with a transferability of 89.19% (Table S1).

## 4. Discussion

In the past decade, as the cost of genomic sequencing has decreased, increasing numbers of genomes from many species have been sequenced and their sequences published. Such data provide a valuable resource to screen and develop genome-wide microsatellites in all types of species, such as microorganisms [27,28], plants [29], and animals [30,31]. The length of sequence reading in third-generation sequencing (e.g., NGS technologies from PacBio or Oxford Nanopore) is longer than that in second-generation sequencing (e.g., NGS technologies from Ion Torrent or Illumina) [32,33]. Therefore, more SSRs can be isolated from the genome sequenced by technologies based on third-generation sequencing. The chromosome level genome of *A. latus* was obtained using a hybrid sequencing strategy with PacBio and Hi-C [21]. These data were useful for the detection of microsatellites in the whole genome of *A. latus*, which lay a foundation for molecular marker-assisted breeding and genetic analyses. Our research is the first comprehensive report on the isolation and analyses of microsatellites in *A. latus*.

SSRs have other desirable properties: high polymorphism, repeatability, codominance, multi-allelic nature, and locus specificity. These properties can be used for analyses of genetic diversity and paternity testing [34]. SNPs in the whole genome can be genotyped readily with high-throughput sequencing, namely “genotyping by sequencing” (GBS) [35]. SNPs are suitable for construction of high-density linkage maps, genome-wide association studies, and genomic breeding [36,37,38]. However, the bi-allelic nature of an SNP marker and lack of transferability across the population hamper efforts by breeders to utilize such information [39]. Therefore, it is necessary to develop the technologies of SSR GBS. Recently, some research on sequence-based microsatellite genotyping has been reported, which may suggest not using SNP genotyping [34,40,41].

A total of 318,862 SSRs were detected from the *A. latus* genome, which is more than that in *Lateolabrax maculatus* (220,709) [18] but less than that in *Hypophthalmichthys molitrix* (368,572) [42]. The total length of SSRs was 9.07 Mb, accounting for 1.32% of the whole genome length (Table S2). The content of SSRs was 1.32% in the *A. latus* genome in the present study, which is higher than that in *H. molitrix* (0.77%) [42] and *L. maculatus* (0.99%) [18] but lower than that in *Danio rerio* (2.31%) [43] and *Gadus morhua* (4.97%) [16]. The percentage of SSRs may be affected by the genome size of species and screening parameters of SSRs [21]. The total length of SSRs is proportional to the genome size in many types of fish [16,18].

Mononucleotide SSRs are neglected as molecular markers because of their instability in PCR amplification [31]. Except for mononucleotide repeats, dinucleotides are the dominant microsatellites in most animals, including fishes [16,18,43], shrimps [44], insects [31], birds [30], and mammals [45,46]. In the present study, the dominant type of SSRs was also dinucleotide (accounting for 76.92%). In general, the instability of dinucleotide repeats is higher than that for other repeats. Therefore, the mutation rates of dinucleotide SSRs are the highest [47]. For instance, the mutation rates of dinucleotides are 1.5–2-times higher than that of tetranucleotides in the human genome [48]. In the present study, the most abundant dinucleotide repeat was (AC)n, followed by (AG)n and (AT)n. A few GC repeats were found because of their high stability [45]. Similar results have been found in mammals, fishes, shrimps, and insects [16,18,31,45].

Trinucleotides, accounting for 15.75%, were the second most abundant repeats in the *A. latus* genome. However, in some species, tetranucleotide was the second dominant repeat [16]. The (AGG)n motif was the predominant trinucleotide type in the *A. latus* genome, which is identical to the situation in *Fugu rubripes* [49], *Eriocheir sinensis* [50], *Ophiophagus hannah* [51], and *Meloidogyne incognita* [52]. In addition, the (AGG)n motif is the dominant type in exons among 10 fish genomes [43]. These results suggest that (AGG)n may have an important role in gene function. (AAT)n is the predominant motif in *L. maculatus* [18], *H. molitrix* [42], and *Monopterus albus* [53]. (AAG)n is the most frequent motif in *Fenneropenaeus chinensis* [54]. (ATC)n is the most frequent motif in the fungus *Puccinia striiformis* f. sp. Tritici [55]. (AAC)n is the more common trinucleotide SSR type in pigs [46]. Thus, the abundance of trinucleotide motifs varies in different species. The number and length of SSRs in a chromosome have been shown to be positively correlated with the size of the chromosome [18,30]. We also confirmed this result (Figure 3).

Most of the SSRs in the *A. latus* genome were found to be in non-coding regions, such as intergenic regions and introns. Only 19,378 SSRs (accounting for 8.55%) were located in exonic regions. These results have also been documented by other scholars [30,31,45]. In addition, more SSRs were detected in introns than in intergenic regions, which has also been investigated in other types of fish [45]. However, the number of SSRs from intergenic regions was greater than that in introns in potatoes [56] and camelids [57]. SSRs located in non-coding regions can affect the function, expression, and transcription of genes [58]. For example, (CT)n located in the 5′ untranslated coding region of IRF can influence the expression of IRF, which has also been used as a molecular marker for resistance selection of *Litopenaeus vannamei* [59,60]. The (AC)_17–39_ repeat located in the promoter region of *HO-1* is related to the development of cancer and Parkinson’s disease [61,62]; The (CA)_17–21_ repeat in the promoter of IGF1 can affect transcription inversely [63]. The (GT)n(GA)m microsatellite located in the DRB1 intron is associated with the growth and reproduction of sheep [64]. Microsatellites located in exonic regions can control gene activation, as well as affect the evolution of protein structure and function [58]. Trinucleotide and hexanucleotides had a propensity to locate in exons (Figure 5), data that aligned with results from other studies [30].

GO analyses showed that genes containing microsatellites were enriched mainly in cellular activities, such as organelles, binding, catalytic activity, biological regulation, and regulation of biological processes. These results are consistent with data from *Ophiophagus hannah* [51]. The expansion of gene families is valuable for phenotypic diversity and the evolutionary adaptions of species [65]. In humans, expansion of microsatellite repeats can cause up to 30 developmental and neurological inherited disorders [66]. Whole-genome duplication could influence the mutational dynamics of SSRs [67]. Zhu et al. [4] compared the gene families of *A. latus* and eight other teleost species and found 238 expanded gene families in *A. latus* (*p* < 0.05). These genes were enriched significantly in some GO terms, which were also observed in our study (Table S4). In addition, the extreme expansion ARRDC3 and SLC12A reported by Zhu et al. [4] was also detected in GO annotations in the present study (Table S4). There results showed that SSRs may be positively related with gene expansion in fish. More investigations are needed to provide a hypothesis.

The genes containing microsatellites were related to immunity and growth and were enriched significantly in KEGG pathways (Figure 7). In pigs, the genes containing SSRs were related to “bone remodeling”, “muscle development”, and “immunity” and are enriched in KEGG pathways [46]. These results suggest that genes containing SSRs may influence the growth and resistance of animals, which could aid the development of new markers for breeding. The transferability rate of SSR markers between *A. latus* and *A. schlegelii* was high (89.19%), which suggested that the relationship between these species was close [68].

## 5. Conclusions

For the first time, many genome-wide microsatellites were isolated and characterized from the *A. latus* genome. The location of microsatellites in exonic, intronic, and intergenic regions was determined, which may lay a foundation to measure how microsatellites influence gene function. More than 217,791 microsatellite markers were developed, which will be beneficial for systematic breeding programs and genetic studies of *A. latus*.

## Figures and Tables

**Table 1:** Summary of SSR repeats in *A. latus* genome.

Repeats	Number	Proportion (%)	Frequency (loci/Mb)	Length (bp)	Density (bp/Mb)
Di-	245,271	76.92%	357.99	7,045,666	10,283.73
Tri-	50,220	15.75%	73.30	1,188,063	1734.08
Tetra-	18,107	5.68%	26.43	628,736	917.69
Penta-	4108	1.29%	6.00	162,205	236.75
Hexa-	1156	0.36%	1.69	45,000	65.68
Total	318,862	100%	468.41	9,069,670	13,237.93

**Table 2:** Top 10 abundant motif categories in genome of *A. latus*.

Categories	Number	Frequency (loci/Mb)	Length (bp)	Density (bp/Mb)
AC	168,390	245.78	5,004,294	7304.18
AG	52,305	76.34	1,113,538	1625.30
AT	24,152	35.25	922,230	1346.07
AGG	15,989	23.34	346,359	505.54
AAT	9198	13.43	292,392	426.77
AAG	6610	9.65	167,997	245.21
AGC	5448	7.95	111,348	162.52
ATC	5069	7.40	113,091	165.07
AAC	4268	6.23	85,422	124.68
AGAT	3038	4.43	144,423	210.80

**Table 3:** The distribution of microsatellites in exon, intron, and intergenic regions of *A. latus*.

Genomic Region	Exons	Intergenic Regions	Introns	All
Di-	7909	45,439	78,991	132,339
Tri-	6655	10,027	14,969	31,651
Tetra-	761	4689	6308	11,758
Penta-	207	1071	1623	2901
Hexa-	70	236	247	553
Compound	3776	16,794	26,801	47,371
All	19,378	78,256	128,939	226,573
Percentage	8.55%	34.54%	56.91%	

**Table 4:** Characteristics of nine polymorphic SSR markers in *A. latus*.

Locus	*Na*	*Ne*	*Ho*	*He*	*I*	*PIC*
Hqd1	8	4.2582	0.1724	0.7786	1.7101	0.7370
Hqd2	10	4.7380	0.3103	0.8028	1.7962	0.7601
Hqd3	9	4.5459	0.1034	0.7937	1.7266	0.7482
Hqd6	6	4.2690	0.4138	0.7792	1.5529	0.7270
Hqd8	4	1.9157	0.5172	0.4864	0.8398	0.4145
Hqd12	3	2.6869	0.4483	0.6388	1.0333	0.5488
Hqd14	8	3.6805	0.1379	0.7411	1.5372	0.6870
Hqd32	7	5.2399	0.2069	0.8234	1.7807	0.7843
Hqd33	7	3.5787	0.1724	0.7332	1.5565	0.6907
mean	6.8889	3.8792	0.2759	0.7308	1.5037	0.6775
St. Dev	2.2608	1.0450	0.1513	0.1064	0.3396	0.1200

Note: *Na*—observed number of alleles; *Ne*—effective number of alleles; *Ho*—observed heterozygosity; *He*—expected heterozygosity; *I*—Shannon’s information index. *PIC*—polymorphism information content.

## Data Availability

Data are contained within the article.

## Supplementary Materials

The following supporting information can be downloaded at: https://www.mdpi.com/article/10.3390/ani14243709/s1, Table S1: Microsatellite markers used in this study; Table S2: The character of SSRs in different chromosomes; Table S3: Summary of SSR motifs and repeats; Table S4: Exon with SSRs enriched in GO terms, which included expanded genes; Table S5: SSR marker counts and proportion of five motifs in each chromosome.
